# Bibliometric Insights and Recent Advances in the Science, Technology, and Sustainability of Açaí (*Euterpe oleracea*) from Amazonian Staple to Global Superfruit

**DOI:** 10.3390/foods15122203

**Published:** 2026-06-18

**Authors:** Adriano Cezar Delphim, Gerson Lopes Teixeira, Adaucto Bellarmino Pereira-Netto

**Affiliations:** 1 Graduate Program in Food Engineering, Department of Chemical Engineering, Federal University of Paraná, Curitiba 82590-300, PR, Brazil; dicoadriano@yahoo.com.br (A.C.D.); adauctodepereira@hotmail.com (A.B.P.-N.); 2 Graduate Program in Food and Nutrition, Federal University of Paraná, Curitiba 80210-170, PR, Brazil

**Keywords:** bioaccessibility, encapsulation, antioxidant activity, food processing, by-product valorization, functional food, circular bioeconomy, bibliometry, Amazonian berry

## Abstract

*Euterpe oleracea* Mart. (açaí), a palm fruit native to the Amazon basin, has attracted growing global scientific interest over the past decade owing to its distinctive phytochemical richness and broad functional potential. This narrative review synthesizes research published between 2015 and 2025 on açaí’s nutritional composition, biological activities, food technological applications, processing innovations, by-product valorization, and sustainability challenges. Açaí pulp contains a distinctive nutrient matrix—including anthocyanins (particularly cyanidin-3-glucoside), polyphenols, oleic and linoleic fatty acids, and dietary fiber—underpinning antioxidant, anti-inflammatory, cardioprotective, hepatoprotective, and antiobesity effects demonstrated primarily in in vitro and animal models, with human clinical evidence still limited. Processing strategies such as ultrasound-assisted extraction, nanoencapsulation, freeze-drying, and supercritical CO_2_ extraction have advanced bioactive stability and bioaccessibility, enabling açaí’s incorporation into dairy products, functional beverages, biodegradable packaging, reformulated meat products, and edible films. Processing residues—seeds and pomace—are increasingly repurposed into nutraceuticals, biosorbents, and bio-based polymers, reinforcing the species’ circular bioeconomy potential. Food safety risks, particularly *Trypanosoma cruzi* contamination in minimally processed products, require standardized mitigation protocols. Key remaining challenges include the absence of validated bioaccessibility methodologies, the scarcity of human clinical trials, and the need for scalable processing technologies suitable for smallholder production contexts. Overall, açaí emerges as a model bioresource at the convergence of nutrition science, food technology, and environmental sustainability.

## 1. Introduction

**Figure 1:**
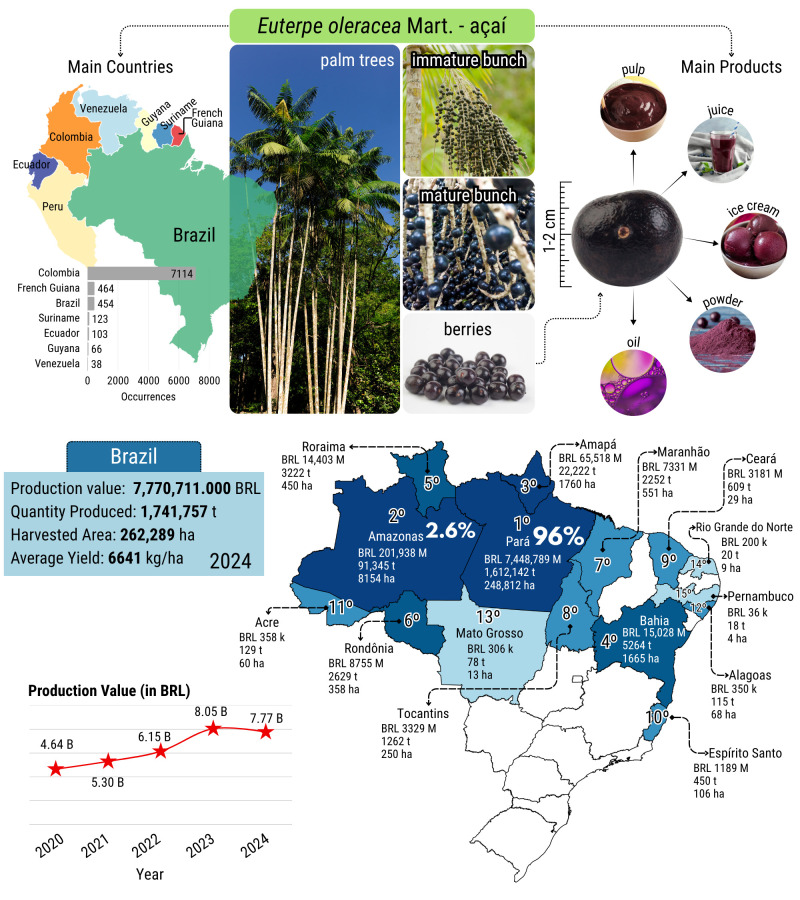
Schematic representation of *Euterpe oleracea* Mart. (açaí), illustrating its distribution in countries with the highest occurrence records, stages of fruit development, principal derived products according to GBIF [1], and Brazilian production statistics for 2024 based on data from IBGE [3]. Images retrieved from Canva PRO database.

*Euterpe oleracea* Mart., known as açaí, a palm species native to the Amazon region, has long been recognized as an important crop, primarily for the food industry. The species occurs naturally in several South American countries, including Brazil, Colombia, French Guiana, Suriname, Ecuador, Guyana, and Venezuela. According to the Global Biodiversity Information Facility (GBIF), 8503 occurrences of *E. oleracea* had been recorded as of 20 May 2026. Although most records originate from Colombia (83.7%) [1], these data represent documented specimens in institutional collections rather than the species’ natural distribution or production geography, where Brazil is the leading producer. Three açaí species have economic potential: *Euterpe oleracea*, *E. precatoria*, and *E. edulis*. *E. oleracea*, native to Pará, is early-bearing; *E. precatoria*, from the Lower Amazon, is productive but late-maturing; and *E. edulis*, from the Atlantic Forest, is used primarily for heart of palm. This study focuses on *E. oleracea*, the most widely produced and consumed species [2]. According to the Brazilian Institute of Geography and Statistics—IBGE [3], açaí is cultivated in 15 Brazilian states, with the largest harvested areas located in Pará (94.86%), Amazonas (3.11%), Amapá (0.67%), Bahia (0.63%), Maranhão (0.21%), Roraima (0.17%), and Rondônia (0.14%). In addition to its edible heart of palm, *E. oleracea* is valued for its small, dark-purple fruits, whose pulp is used to prepare a beverage traditionally known in Brazil as “açaí wine.” Some characteristics, recorded occurrences, main products, and production statistics related to açaí are illustrated in Figure 1.

Brazil is the world’s leading producer of açaí, accounting for approximately 90–95% of global açaí berry production in 2025. Consistent with IBGE [3], the production value in 2024 reached BRL 7.77 billion (approximately USD 1.50 billion, based on an exchange rate of 1 USD = 5.17 BRL, as provided by the Brazilian Central Bank currency conversion tool [https://www.bcb.gov.br/en/currencyconversion], accessed on 9 June 2026), with 1.742 million metric tons harvested from 262,289 hectares, corresponding to an average yield of 6641 kg/ha. The state of Pará overwhelmingly dominates national production, accounting for approximately 96% of the total output, with a production value of BRL 7.45 billion (approximately USD 1.44 billion), followed by Amazonas (BRL 201.9 million) and Amapá (BRL 65.52 million). The remaining 12 producing states collectively contribute about 1% of national production (Figure 1).

Future Market Insights [4] estimates the global açaí berry market will grow from USD 10,006.8 million in 2026 to USD 23,359.8 million by 2036 (CAGR 8.8%), with raw/whole açaí berries holding a 56.4% share. Growth is especially strong in emerging economies such as India (CAGR 13%) and Thailand (12%), followed by the USA (CAGR 8.2%). The report highlights key drivers, including increasing use in smoothies, energy drinks, and supplements, brand expansion, and market development in Europe and Asia. It also points to opportunities in pharmaceutical and nutraceutical applications, underscoring açaí’s potential in preventive health and therapeutic products.

Açaí’s versatility spans a wide range of industries, particularly in food and beverages, where it appears in bakery items, confectionery, infant formulas, snacks, desserts, juices, nectars, squashes, and smoothies. Beyond food, açaí is incorporated into dietary supplements, cosmetics, personal care products, and pharmaceuticals, highlighting its broad appeal in health, nutrition, and wellness markets [4]. Globally recognized as a “superfruit,” açaí’s broad applications in the food and beverage industry are largely attributable to its nutrient-dense composition, which varies according to the processing method applied [5,6,7]. Açaí pulp contains substantial carbohydrates (31.82–56.83 g 100 g^−1^ in fresh pulp; 82.48 g 100 g^−1^ in spray-dried) and lipids (33.52–53.31 g 100 g^−1^), relatively high protein (3.83–10.67 g 100 g^−1^), and low ash content (1.62–4.20 g 100 g^−1^) [6]. Beyond macronutrients, açaí is an abundant source of bioactive compounds that enhance its functional food potential, which includes high levels of anthocyanins (particularly cyanidin-3-glucoside), flavonoids, dietary fiber, unsaturated fatty acids (notably oleic and linoleic), vitamins, and essential minerals. These constituents have been linked to a broad spectrum of biological activities, including antioxidant, anti-inflammatory, cardioprotective, neuroprotective, and anticarcinogenic effects, as demonstrated in both in vitro studies and animal models [8,9,10,11,12].

The nutrient-dense composition and sensory appeal of açaí make it a highly promising ingredient across a wide range of product categories, from functional foods to nutraceuticals. Technological research has expanded to diverse food matrices, including dairy desserts [5], probiotic yogurt [13], edible films and coats [14,15], sauce [16], snacks [17], ice cream and cookies [18], burgers [19,20], as well as pork patties [21], to combine health benefits with sensory appeal. At the same time, technological strategies such as freeze-drying, cold plasma, nanoencapsulation, ultrasound-assisted extraction, and supercritical CO_2_ extraction have been employed to preserve or enhance the stability and bioavailability of açaí’s functional compounds during processing and storage [7,22,23,24,25,26,27].

Despite the growing body of research, several knowledge gaps remain in the literature. Although numerous studies have explored specific applications and bioactivities of açaí, reviews that critically map the evolution, collaboration networks, and thematic focus areas of this research domain are lacking. Here we present a narrative review of research on açaí in food sciences over the past decade. Using mapping of publication trends, leading institutions, co-authorship networks, and thematic clusters, this article seeks to synthesize current knowledge and identify future opportunities in the science and technology of this emblematic Amazonian species, açaí.

## 2. Methodology

The primary strategy used for article retrieval is presented in Figure S1, adapted from the PRISMA 2020 statement [28]. Searches were initially conducted in both the Scopus and Web of Science Core Collection (WoS) databases; however, WoS was selected as the primary database due to its higher document yield and broader coverage of the topic. On June 8, 2026, an advanced search was performed in WoS using the query TS = (“*Euterpe oleracea*” OR “açaí” OR “acai” OR “assai”), retrieving 2220 records. The screening and eligibility procedures followed the PRISMA workflow. First, the publication period was restricted to studies published between 2015 and 2025, resulting in 1515 records. Subsequently, non-research publication types were excluded, retaining only original research articles (*n* = 1236). Further refinement for “food” using the WoS “search within results” tool reduced the dataset to 466 records. Finally, titles and abstracts were manually screened for eligibility, resulting in the inclusion of 245 studies in the final dataset. Studies were excluded if they focused exclusively on non-food matrices or on açaí by-products and residues (leaves, seeds, kernels, extraction cake, pomace, and other waste materials), or if they were unrelated to food applications, processing, or bioactive characterization. Clinical studies on açaí consumption have been comprehensively covered in recent reviews [29,30,31] and were therefore excluded from the present analysis. This review accordingly focuses on mechanistic, in vitro, and animal studies. Bibliometric analyses were conducted using VOSviewer (v. 1.6.20) and R software (v. 4.3.3) through the Bibliometrix package (v. 4.3.0) [32,33]. Selected datasets were additionally exported to Microsoft Excel for data organization and graphical processing in OriginPro (v. 8.6).

## 3. Relevance of *Euterpe oleracea* Species

**Figure 2:**
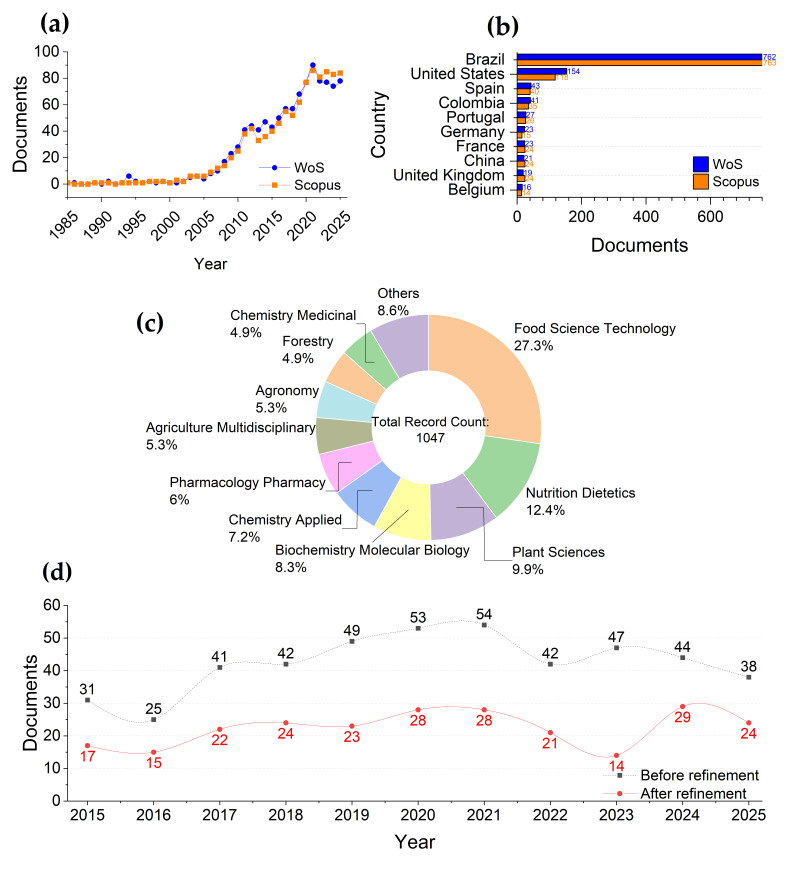
Comparison of documents indexed in Scopus and Web of Science (WoS) related to *E. oleracea* from 1985–2025 (**a**); leading publishing countries (**b**); main WoS research areas (**c**); and publication trends for *E. oleracea* and “food” in the WoS Core Collection from 2015–2025 before (dashed line) and after (solid line) exclusion of unrelated articles (**d**).

Studies on *E. oleracea* were first published in 1985, and scholarly interest has steadily grown since 2000, as evidenced by the data in Figure 2a. Brazil is the predominant contributor to the research, with 696 documents in WoS (71.9%) and 694 in Scopus (74.1%). The United States ranked second, with 150 articles in WoS (15.5%) and 114 in Scopus (12.2%). These data confirm Brazil’s central role in advancing the understanding of *E. oleracea*, followed by the USA and, to a lesser extent, by countries such as Spain and Colombia (Figure 2b).

According to the WoS platform, most studies on *E. oleracea* are concentrated in the field of *Food Science and Technology*, which accounts for 27.3% of the total research output. This is followed by *Nutrition and Dietetics* (12.4%) and *Plant Sciences* (9.9%), reflecting the species’ importance in both nutritional and agricultural contexts. Other prominent research areas include *Biochemistry and Molecular Biology* (8.3%), *Applied Chemistry* (7.2%), *Pharmacology and Pharmacy* (6%), and *Agriculture Multidisciplinary* (5.3%) (Figure 2c). These findings highlight the broad multidisciplinary interest surrounding *E. oleracea*, with a clear emphasis on its potential as a functional food and its various applications in health, chemistry, and agriculture. This diversity of research areas underscores the importance of the species in numerous scientific domains, emphasizing its relevance beyond just the food and nutrition sectors.

Although WoS and Scopus provided similar results, the former was chosen for the bibliometric analysis of *E. oleracea* because it retrieved a greater number of relevant documents. The initial keyword search identified 466 articles; however, after applying refinement criteria to exclude unrelated studies, 245 publications were retained for analysis. As illustrated in Figure 2d, the number of publications on açaí-based food products increased steadily beginning in 2015, reaching a peak of 28 publications in 2021. This was followed by a decline to 14 publications in 2023, before partially recovering to 29 publications in 2024.

## 4. Bibliometric Insights into Açaí Research

As shown in Table S1, a total of 1336 authors contributed to the literature on açaí, with an average of 6.91 co-authors per document and no single-authored publications. International co-authorship represented 23.67% of the documents, highlighting the globally interconnected nature of the research network. The scientific output was distributed across 124 sources, reflecting the thematic diversity of contributing journals. The annual growth rate of scientific production was 3.51%, indicating a sustained research interest despite disruptions caused by the COVID-19 pandemic [34]. The field exhibited substantial keyword diversity, with 810 author keywords covering major themes such as antioxidant capacity, anthocyanins, bioactive compounds, and technological processing. Documents averaged 5.73 years of age and 17.73 citations each, suggesting continued relevance of the core literature.

### 4.1. Keyword Co-Occurrence Analysis

**Figure 3:**
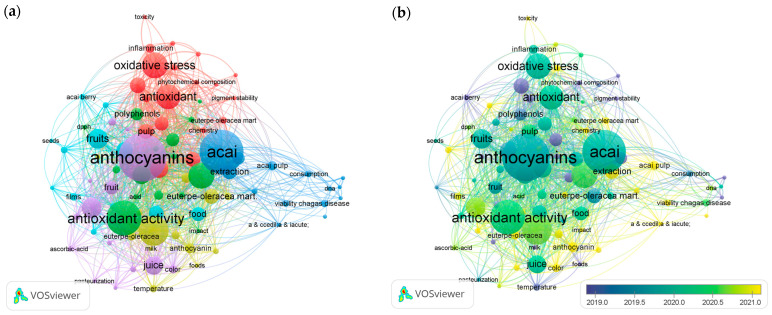
Keyword clusters for *Euterpe oleracea* (açaí) berry products research articles, colored by topic similarity (**a**) or publication year (**b**). Data retrieved from WoS Core Collection from 2015 to 2025 and analyzed by VOSviewer.

A keyword co-occurrence analysis was conducted using VOSviewer to identify research trends and thematic clusters within the literature on *E. oleracea* berry products. According to van Eck & Waltman [32], VOSviewer visually represents keywords based on their frequency and co-occurrence within the dataset. Larger clusters indicate more frequently occurring keywords across the studies, while the color grouping reflects thematic relationships among keywords. In this analysis, six distinct clusters emerged, containing 12–19 keywords each and representing different research directions related to the bioactive properties and applications of açaí and its derivatives (Table S2). Figure 3 illustrates the main keyword clusters identified in research articles on *E. oleracea* (açaí) berry products, retrieved from WoS Core Collection from 2015 to 2025. Figure 3a colors clusters by keyword similarity, while Figure 3b shows them by publication year, with greenish representing older studies and yellowish for newer ones.

Cluster 1 groups terms such as antioxidant, oxidative stress, obesity, inflammation, and phytochemical compounds, together with experimental models such as rats, indicating a biomedical focus on the phytochemical profile of açaí and its potential role in chronic disease modulation. Cluster 2 includes keywords such as phenolic compounds, polyphenols, antioxidant activity, and DPPH, highlighting studies focused on antioxidant characterization and analytical evaluation of bioactive compounds. Cluster 3 is associated with food safety and contamination issues, featuring terms such as Chagas disease, *Trypanosoma cruzi*, oral transmission, and outbreak, reflecting concerns regarding the consumption of contaminated raw açaí products. Cluster 4 emphasizes compositional and technological aspects, including açaí oil, anthocyanin, phenolic compounds, stability, optimization, and physical properties, often related to food applications and product development. Cluster 5 is primarily associated with processing and quality evaluation, with keywords such as anthocyanins, pasteurization, thermal processing, color, physicochemical properties, and total antioxidant capacity. Cluster 6 reflects emerging approaches involving bioaccessibility, encapsulation, films, antioxidant properties, and seed utilization, indicating growing interest in by-product valorization and functional delivery systems. This multifocal structure underscores the interdisciplinary nature of açaí research, bridging food science, pharmacology, and public health.

**Figure 4:**
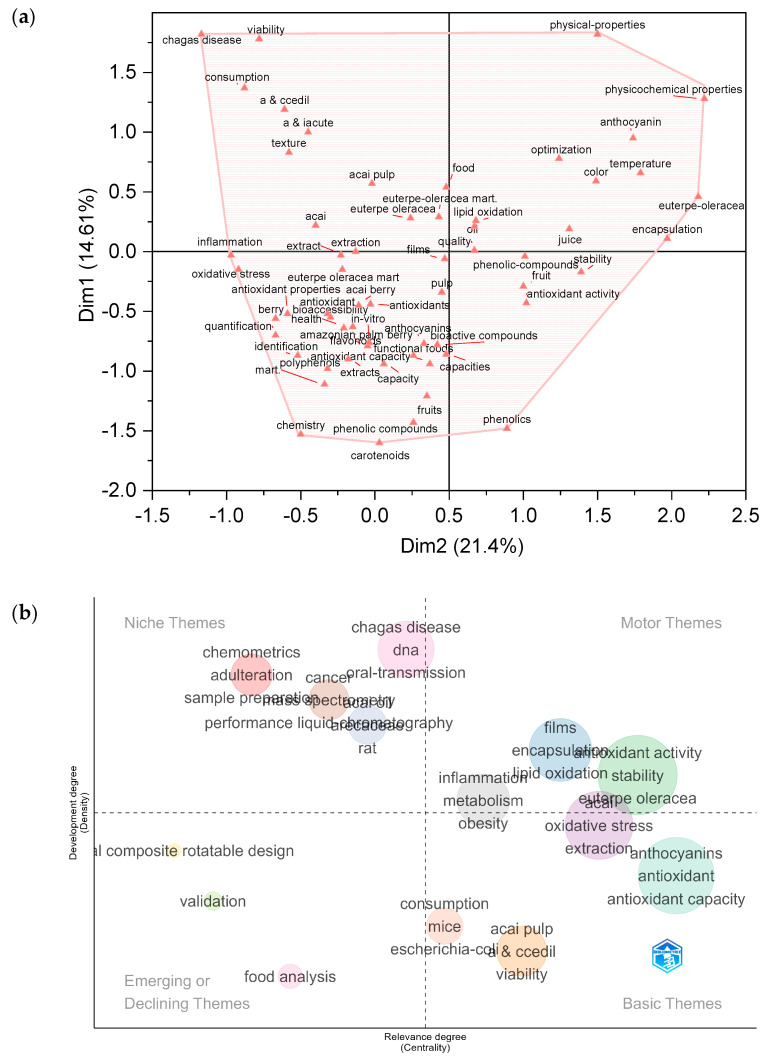
Conceptual structure map showing main keywords related to *Euterpe oleracea* (açaí) berry products from Multiple Correspondence Analysis—MCA (**a**), and a thematic map depicting niche, motor, emerging/declining, and basic themes (**b**) as performed in Bibliometrix analysis using data from the WoS Core Collection.

Figure 4 illustrates the conceptual structure and thematic organization of açaí research based on author keyword co-occurrence and multiple correspondence analysis (MCA).

The MCA plot (Figure 4a) reveals two main research orientations. One cluster is associated with biomedical studies, including keywords such as oxidative stress, inflammation, and antioxidant properties, reflecting investigations into the health effects of açaí bioactive compounds. The second cluster relates to food science and technology, with terms such as encapsulation, physicochemical properties, temperature, and stability, indicating efforts to improve processing, preservation, and functional food applications. Additional terms such as viability, consumption, and Chagas disease reflect specific public health and microbiological safety concerns linked to açaí products. The thematic map (Figure 4b) further classifies these topics according to their development and relevance. Motor themes, including *antioxidant activity*, *stability*, and *Euterpe oleracea*, represent well-developed and influential research directions. Basic themes, such as *anthocyanins* and *antioxidant capacity*, remain central to the field but require further development. Niche themes, including *Chagas disease* and *DNA*, correspond to specialized biomedical investigations, while emerging or declining themes, such as *food analysis* and experimental design approaches (e.g., *central composite rotatable design*), suggest methodological developments or less consolidated research lines.

Analyses of the conceptual structure map demonstrate that açaí research is anchored in its antioxidant and phenolic composition, with strong emphasis on health-promoting bioactivity. It also demonstrates that research on açaí is expanding into disease prevention, compound stabilization, and advanced food processing strategies, which reflects a convergence between biomedical and technological innovation.

### 4.2. Leading Research Institutions

The analysis of leading institutions in açaí research revealed complementary perspectives depending on the bibliometric tool applied. Bibliometrix (Figure S2), which consolidates institutional name variants, identified Federal University of Pará (UFPA) as the leading institution with 55 publications, followed by State University of Campinas (UNICAMP) with 52 publications. University of São Paulo (USP) and Brazilian Agricultural Research Corporation (EMBRAPA) followed with 31 and 27 publications, respectively.

In contrast, VOSviewer does not merge institutional variants, resulting in multiple entries for the same institution. For example, UNICAMP appeared as both “Univ Estadual Campinas” (21 documents, 506 citations, TLS 10) and “Univ Campinas Unicamp” (10 documents, 165 citations, TLS 4). When combined, these records highlight UNICAMP as a major contributor to açaí research. USP ranked second, with 18 documents, 253 citations, and the highest TLS (13), reinforcing its central role in collaborative networks (Table S3). Other important Brazilian institutions included UFC (17 documents, 421 citations) and UFV (13 documents, 328 citations), with notable contributions to antioxidant studies, extraction processes, and food technology applications.

Differences in institutional publication counts between Bibliometrix and VOSviewer reflect their distinct approaches to affiliation extraction and normalization. Bibliometrix systematically standardizes and aggregates institutional name variants, whereas VOSviewer relies on raw affiliation strings, which may retain residual naming variation. Absolute counts from the two tools should therefore not be interpreted as directly equivalent, although both consistently identify UNICAMP as a leading contributor.

VOSviewer also highlighted international participation, led by Texas A&M University (8 documents, 257 citations), followed by the University of Reading and the Warsaw University of Life Sciences, reflecting the growing global interest in the nutritional and technological potential of açaí.

### 4.3. Geographic Distribution and International Collaboration on Açaí Research

Table 1 reveals a clear geographic concentration of research on *E. oleracea*, overwhelmingly led by Brazil, which accounts for 192 of 245 publications (over 78%) between 2015 and 2025. Brazil also shows the highest number of citations (3238) and total link strength (TLS = 38), reaffirming its central role in açaí research due to the fruit’s native distribution in the Amazon basin and its deep integration into Brazilian agriculture and culture.

Following Brazil, the United States ranks second, with a smaller number of papers but a higher citation-per-document (C/D) ratio (19.74 vs. 16.86 for Brazil), underscoring the global influence of North American studies that often focus on clinical trials, bioavailability, and health-related applications. Strong collaborative links (TLS = 18) between U.S. and Brazilian institutions further highlight the importance of transcontinental cooperation in this field. Within Latin America, Colombia and Chile also stand out, with 5 and 3 documents and C/D ratios of 20.60 and 15.67, respectively, reflecting regional interest in leveraging Amazonian biodiversity for value-added food and nutraceutical products.

Across Europe, countries such as Poland, Spain, England, and Germany contribute actively to the field, particularly in food technology, analytical chemistry, and nutrition. Portugal, despite a lower publication count, achieves the highest citation-per-document ratio (C/D = 60.50), likely reflecting the strong scientific and linguistic ties between Portuguese and Brazilian researchers.

Asian countries, including South Korea, China, and Türkiye, represent an emerging front in açaí research. Notably, China has accumulated 130 citations from just four papers, reflecting its growing influence through high-impact publications. The participation of these newer contributors, along with Eastern European partners, signals an expanding research agenda that now encompasses functional foods, nutraceuticals, and pharmaceutical applications of açaí.

**Figure 5:**
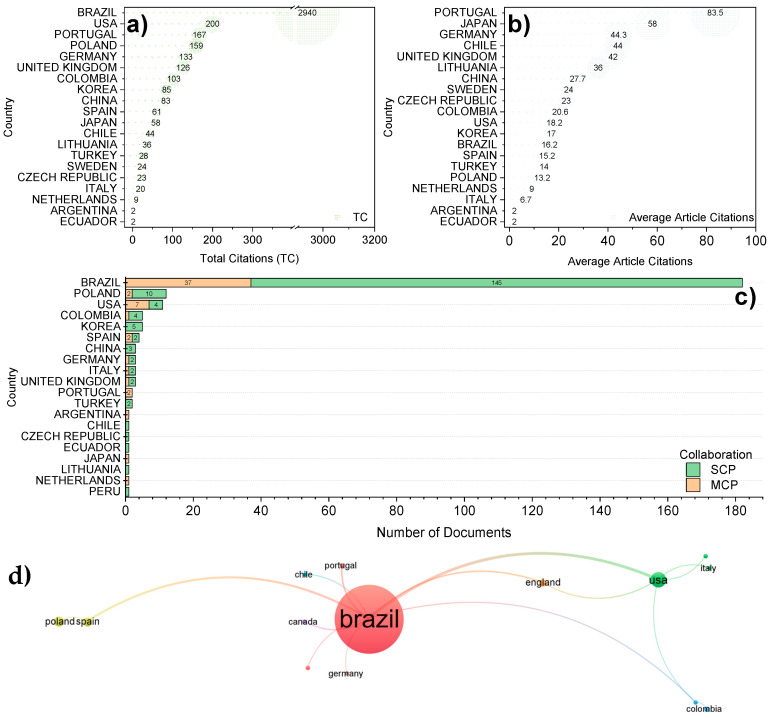
Bibliometrix ranking of countries publishing research on açaí, based on total citations (**a**), average article citations (**b**), and collaboration (single-country publications versus multiple country publications) (**c**), along with VOSviewer analysis of international collaboration clusters among countries involved in research on açaí from 2015 to 2025 (**d**).

Despite slight variations between tools, *Bibliometrix* results corroborate Brazil’s leadership (2940 citations), followed by the USA (200), Portugal (167), and Germany (133) (Figure 5a). However, when citation impact per publication is considered, a different picture emerges: Portugal, Japan, and Germany lead with 83.5, 58, and 44.3 citations per article, respectively. This pattern suggests that while Brazil dominates in output, smaller contributors often produce more targeted, high-impact studies, potentially reflecting specialized collaborations or focused thematic niches (Figure 5b). The comparatively lower average citation rate for Brazilian studies (16.2 citations/article) may be partially attributed to the prevalence of Portuguese-language publications and non-indexed national journals, which reduce international visibility. Remarkably, our study only included English-language articles published in WOS-indexed journals, meaning many Brazilian studies were excluded from the analysis.

Analysis of international collaboration, measured through Multiple Country Publications (MCP) and Single Country Publications (SCP), further refines this picture (Figure 5c). Brazil remains the main research hub but conducts only 20.3% of its work through international partnerships, reflecting a largely domestic research model supported by local access to biological material and national funding programs. Conversely, smaller-output countries, such as Portugal, Argentina, Japan, the Netherlands, and Sweden, rely heavily on international collaboration, with most of their publications produced through joint partnerships—typically with Brazilian institutions. The collaboration network (Figure 5d) confirms Brazil as the central hub in global açaí research. The strongest collaboration occurs between Brazil and the United States, forming the main axis of international cooperation. Additional connections link these countries with Spain, Poland, Portugal, Canada, Chile, and England, forming secondary collaborative clusters. Smaller nodes such as Colombia and Italy indicate emerging or more specialized research contributions.

### 4.4. Leading Sources Publishing Açaí Research

**Figure 6:**
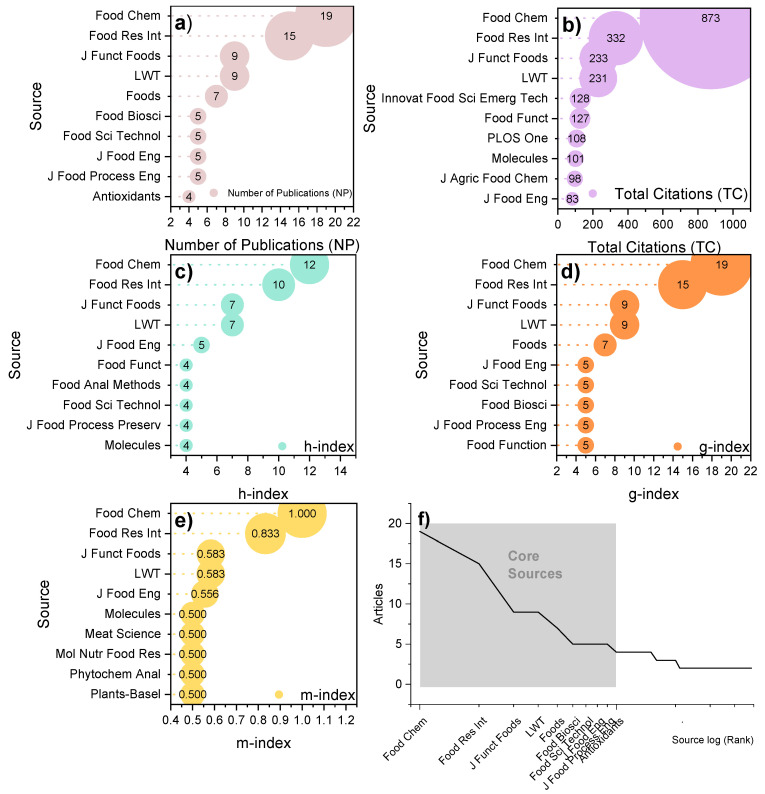
Journals rankings from studies on *Euterpe oleracea* berry products based on the number of publications (**a**), total citations (**b**), h-index (**c**), g-index (**d**), m-index (**e**), and the core sources according to Bradford’s Law (**f**), as determined by Bibliometrix analysis with data from the WoS Core Collection.

The analysis of source productivity and influence (Figure 6) offers a comprehensive overview of the journals shaping the scientific landscape of açaí research between 2015 and 2025, based on the Web of Science Core Collection and Bibliometrix analysis.

Food Chemistry stands out as the leading publication venue, accounting for 19 articles, followed by Food Research International (15), and the Journal of Functional Foods (9), demonstrating that açaí research is strongly concentrated in high-impact journals within the food science domain (Figure 6a). In terms of total citations (Figure 6b), Food Chemistry also leads with 873, far surpassing Food Research International (332) and the Journal of Functional Foods (233). Together, these results confirm Food Chemistry as the primary dissemination channel for açaí research, combining both volume and visibility in the global scientific community.

When research influence is evaluated through h-, g-, and m-index metrics (Figure 6c–e), Food Chemistry maintains its leading position (h = 12; g = 19; m = 1), indicating consistent and long-term scholarly impact. LWT—Food Science and Technology, Food Research International, and Journal of Functional Foods also demonstrate solid performance, consolidating a strong secondary tier of journals that balance productivity and citation strength. This trend indicates an increasing alignment between açaí research and specialized journals emphasizing food chemistry, metabolomics, and bioactive characterization. Application of Bradford’s Law (Figure 6f) further confirms that the majority of impactful papers are concentrated within a core group of journals—Food Chemistry, Food Research International, Journal of Functional Foods, LWT, and Foods. Beyond this cluster, productivity and citation impact decline sharply, suggesting that açaí research remains highly specialized yet well-positioned within high-impact food science platforms.

### 4.5. Top-Cited Articles in Açaí Research (2015–2025)

The most influential studies on açaí research, summarized in Table 2, collectively illustrate the evolution of the field from basic compositional characterization to functional validation, as well as applied technological and biomedical research.

Early analytical works by Paz et al. [35] and Bataglion et al. [36] established the chemical and antioxidant benchmarks for açaí, particularly regarding its phenolic and anthocyanin profiles. Their methodological rigor set reference standards for authentication and quality assessment of açaí-based products and other similar products, contributing to the fruit’s recognition as a functional food. Building on this foundation, Garzón and co-workers [37] in their study about Colombian açaí expanded the understanding of geographic and environmental influences on polyphenol variability, an important consideration for standardization and labeling of commercial products.

Subsequent investigations have increasingly emphasized health-related and processing-oriented perspectives. Preclinical studies, such as those by Oliveira et al. [11], provided mechanistic evidence of açaí’s capacity to modulate lipid metabolism, inflammation, and oxidative stress, reinforcing its relevance in metabolic health research. Complementing these findings, Peixoto et al. [38] evaluated the effects of *Euterpe precatoria* açaí on aging-related pathways in *Caenorhabditis elegans*, demonstrating that it can prolong lifespan and enhance stress resistance—contributing to a better understanding of açaí’s potential benefits in human longevity research.

More recent investigations have emphasized technological and processing aspects. Dantas et al. [39] and Silveira et al. [40] demonstrated that both gastrointestinal digestion and high-pressure processing substantially influence the stability, bioaccessibility, and antioxidant capacity of açaí compounds, bridging the gap between functional potential and industrial feasibility. Likewise, Neves et al. [41] examined the degradation of bioactives during fruit ripening, informing post-harvest and supply chain optimization strategies.

**Table 2:** Ranking of top articles in açaí research (2015–2025) based on Web of Science analysis of document count.

#	Article	Journal	Year	Aim	Results	TC	TC/Year	NTC	Ref.
1	Brazilian fruit pulps as functional foods and additives: Evaluation of bioactive compounds	Food Chemistry	2015	To analyze the bioactive compound profile (polyphenols, anthocyanins, carotenoids) and antioxidant capacity of Brazilian fruit pulps, including açaí.	Açaí exhibited the highest antioxidant capacity and anthocyanin content, reinforcing its potential as a functional food.	152	12.67	3.50	[35]
2	Determination of the phenolic composition from Brazilian tropical fruits by UHPLC–MS/MS	Food Chemistry	2015	To identify and quantify phenolic compounds in Brazilian tropical fruits using UHPLC–MS/MS.	Açaí showed a diverse profile of flavonoids and phenolic acids; the method proved sensitive and suitable for functional food characterization.	125	10.42	2.88	[36]
3	Polyphenolic composition and antioxidant activity of açai (*Euterpe oleracea* Mart.) from Colombia	Food Chemistry	2017	To evaluate the polyphenolic content and antioxidant activity of Colombian-grown açaí.	The fruit showed high cyanidin derivatives and strong radical scavenging activity, comparable to Brazilian açaí.	92	9.20	3.68	[37]
4	*Euterpe oleracea* Mart.-derived polyphenols protect mice from diet-induced obesity and fatty liver by regulating hepatic lipogenesis and cholesterol excretion	PLoS ONE	2015	To test the effect of açaí polyphenols on liver lipid metabolism and obesity in mice.	Supplementation reduced hepatic steatosis, improved lipid excretion, and modulated gene expression related to fat accumulation.	88	7.33	2.02	[11]
5	Bioaccessibility of phenolic compounds in native and exotic frozen pulps explored in Brazil using a digestion model coupled with a simulated intestinal barrier	Food Chemistry	2019	To evaluate the digestion stability and intestinal bioaccessibility of phenolics from native and exotic fruit pulps, including açaí.	Açaí showed high phenolic content, but only partial bioaccessibility post-digestion, suggesting the need for protective delivery systems.	86	10.75	3.42	[39]
6	An anthocyanin-rich extract of acai (*Euterpe precatoria* Mart.) increases stress resistance and retards aging-related markers in *Caenorhabditis elegans*	Journal of Agricultural and Food Chemistry	2016	To investigate whether an anthocyanin-rich extract of açaí can promote longevity in *Caenorhabditis elegans*.	The extract increased lifespan, enhanced oxidative stress resistance, and reduced age-related markers.	85	7.73	2.77	[38]
7	Anthocyanins, non-anthocyanin phenolics, tocopherols and antioxidant capacity of açaí juice (*Euterpe oleracea*) as affected by high pressure processing and thermal pasteurization	Innovative Food Science & Emerging Technologies	2019	To evaluate the impact of high-pressure and thermal pasteurization on bioactive compound retention in açaí juice.	High-pressure processing preserved anthocyanins and antioxidant activity more effectively than thermal treatment.	70	8.75	2.79	[40]
8	Post-harvest nutraceutical behaviour during ripening and senescence of 8 highly perishable fruit species from the Northern Brazilian Amazon region	Food Chemistry	2015	To monitor antioxidant and phenolic content changes in Amazonian fruits, including açaí, during ripening and senescence.	Açaí’s antioxidant levels peaked early in ripening, declining rapidly in late stages.	66	5.50	1.52	[41]
9	Supercritical CO2 extraction of açaí (*Euterpe oleracea*) berry oil: Global yield, fatty acids, allelopathic activities, and determination of phenolic and anthocyanins total compounds in the residual pulp	The Journal of Supercritical Fluids	2016	To evaluate the supercritical CO_2_ extraction of lyophilized açaí pulp, determining global yield, fatty acid profile, allelopathic activity, and phenolic/anthocyanin contents in the post-extraction residue.	Highest yield at 70 °C/490 bar; lipid profile dominated by MUFA (oleic acid) over PUFA; phytotoxic effect on Mimosa pudica; and increased phenolic and anthocyanin contents in the residual pulp after CO_2_-SE, indicating nutraceutical potential.	62	5.64	2.02	[42]
10	Formulation and characterization of water-in-oil nanoemulsions loaded with açaí berry anthocyanins: Insights of degradation kinetics and stability evaluation of anthocyanins and nanoemulsions	Food Research International	2018	To formulate and characterize water-in-oil (W/O) nanoemulsions encapsulating anthocyanin-rich açaí berry extract, evaluating physical and chemical stability as well as degradation kinetics.	Nanoemulsions remained stable for 30 days at 4 °C with no phase separation; anthocyanin retention followed first-order kinetics; estimated half-life of 385 days for the 2% AE at φd 10 wt% formulation, demonstrating high protective efficiency of the W/O system.	58	6.44	2.05	[43]

Citations based on the Web of Science Core Collection (as of 31 December 2025), from Bibliometrix results. TC, Total Citations; NTC, Normalized Total Citations; Ref., reference.

The studies compiled in Table 2 reveal several convergent patterns. First, antioxidant capacity and anthocyanin content emerge as the most consistently reported endpoints across compositional studies [35,36,37], reflecting their established role as markers of açaí quality and functional potential. Second, a progressive methodological shift is evident: early studies relied primarily on spectrophotometric antioxidant assays and HPLC-based profiling, whereas more recent top-cited works employ simulated digestion models [39], high-pressure processing [40], and in vivo murine models [11], signaling the field’s maturation toward functional validation. Third, processing is a critical determinant of bioactive compound fate: both gastrointestinal digestion [39] and thermal or pressure treatments [40] substantially reduce or preserve bioactives depending on the method applied —a finding with direct implications for product development. Fourth, despite the diversity of approaches, a recurring limitation across these studies is the predominance of in vitro and animal-model evidence, with human clinical data still largely absent, constraining the translation of observed effects into dietary recommendations. Finally, the geographic expansion of açaí research—from Brazilian-centric analyses [35,36] to Colombian growing conditions [37] and advanced encapsulation systems [43]—reflects the fruit’s growing global scientific relevance and the need for standardized methodologies to allow cross-study comparisons.

### 4.6. Trends in Açaí Research

The analysis of trending topics (Figure S3) illustrates the temporal evolution of research themes on açaí, revealing how scientific focus has shifted from fundamental biochemical studies to applied technological and health-oriented investigations. During the early phase (2015–2017), research was primarily exploratory, emphasizing in vitro assays and general antioxidant mechanisms, as demonstrated by Costa et al. [44] and Shim et al. [45], which laid the biochemical foundation for later applied research. Between 2017 and 2018, the emergence of keywords such as fruits, juice, and anthocyanins reflected growing interest in product development, stability, and compound characterization, as highlighted by Garzón et al. [37] and Carvalho et al. [46].

From 2018 to 2020, açaí research underwent a clear thematic expansion toward functionality and technological applications, emphasizing antioxidant activity, stability, and process optimization. This period marked the transition from compositional analysis to food processing and preservation strategies [8,40,47,48,49]. Concurrently, biomedical-related topics such as inflammation and metabolic regulation gained prominence, with studies by Pirozzi et al. [50] and Contente et al. [51] underscoring the relevance of açaí’s bioactives in disease prevention and therapy.

In the most recent period (2021–2025), emerging keywords such as texture, physical properties, and antioxidant capacity highlight an increasing convergence between food engineering, sensory science, and functional nutrition, as explored by Dantas et al. [52], Oliveira et al. [53], and Santos et al. [54] Additionally, research on technological innovations to improve the composition and stability of açaí products has flourished [55,56,57,58,59]. This evolving thematic landscape reflects a broader shift from fundamental biochemical studies to integrative approaches that combine composition, bioactivity, processing optimization, and product quality, addressing both academic research and industrial advancement.

From a product development perspective, Silveira et al. [6] noted that açaí has expanded far beyond its traditional consumption in Brazil, becoming a versatile ingredient used in a wide array of food and beverage products globally. Commercial market now offers açaí in various forms, ranging from energy drinks and juices to teas, electrolyte drinks, kombucha, and even alcoholic beverages. In the dessert industry, açaí appears in sorbets, chocolates, jams, and creamy preparations, while functional products include protein powders, shakes, and concentrated “shots.” Dairy derivatives such as ice creams, yogurts, and frozen desserts, along with more unconventional applications like açaí-based flour, tapioca, and even pepper, further underscore its versatility. In cereals and snacks, açaí is incorporated into bars, toaster pastries, cereals, popcorn, and purées, reflecting its appeal across both health-conscious and indulgence-driven markets.

Recent industry analyses corroborate these bibliometric findings. The Future Market Insights 2025 Report [4] emphasizes that digitalization, AI-driven processing, and predictive agricultural technologies are transforming açaí supply chains, improving efficiency and scalability. Similarly, the 2025 Grand View Research Report [60] reinforces the robust growth of the global açaí berry market, with food and beverage applications continuing to dominate, particularly through the use of the fruit in smoothies, fresh, and functional drinks. The cosmetics segment, however, stands out as the fastest-growing sector, driven by açaí’s antioxidant-rich profile and increasing consumer demand for natural skincare ingredients. While specific figures were not disclosed, the report emphasizes a clear preference for açaí pulp over dried forms, reflecting a growing trend towards minimally processed, nutrient-dense products. Additionally, emerging opportunities in pharmaceutical and nutraceutical applications are also mentioned, underscoring açaí’s expanding role in preventive health and therapeutic uses.

## 5. Current Findings in Açaí Research

Table 3 compiles recent studies on açaí (as of 31 December 2025), revealing a dynamic and multidisciplinary research landscape. Most contributions originate from Brazil, reaffirming the central role of the country as the natural source hub for açaí research. Nevertheless, contributions from the United States, Colombia, and Türkiye indicate a growing internationalization of research on açaí’s nutritional, technological, and biomedical potential.

The majority of recent studies fall within Food Technology, although Health Sciences and Animal Sciences are also strongly represented. Publications on açaí appear in high-impact factor international journals such as *Food Bioscience*, *ACS Food Science & Technology*, *Meat Science*, *Antioxidants*, *Journal of Ethnopharmacology*, *Applied Sciences*, *Tropical Animal Health and Production*, and *Veterinary Research Communications*, highlighting the multidisciplinary reach of açaí.

Overall, recent studies demonstrate that açaí is no longer viewed merely as a “superfruit,” but as a versatile research platform driven by its rich content of bioactive metabolites. Applications now extend beyond nutrition to encompass functional foods, animal feed, advanced processing technologies, active packaging, and emerging biomedical uses. This diversification highlights a significant shift from descriptive characterization to applied innovation; however, the translation of laboratory findings into safe, clinically validated health applications remains an ongoing challenge that requires rigorous human evidence.

Regarding Food Technology, recent studies on açaí have focused on innovations in the use of emerging extraction techniques, the development of bioactive-enriched products, sustainability, and by-product valorization. For example, Bordulis et al. [61] demonstrated that incorporating açaí pulp and seed flour into functional shakes for the elderly not only improved antioxidant activity but also enhanced fiber content of the shakes, providing dual benefits for aging populations while simultaneously valorizing agro-industrial byproducts. Gamarra-Castillo et al. [18] characterized the nutritional composition and antioxidant properties of Colombian açaí and optimized anthocyanin extraction using ultrasound-assisted extraction (UAE). Colombian açaí was found to be rich in lipids, protein, dietary fiber, and essential minerals, while also exhibiting strong antioxidant activity. The study also highlighted the potential of açaí in food product development, particularly in cookies and ice cream, both of which received high acceptability. However, stability issues were observed in the ice cream, attributed to protein precipitation caused by milk acidification, resulting from increased acid levels introduced by the açaí extract.

Rodríguez et al. [62,63] published two complementary studies characterizing four underexplored Amazonian fruits from the Colombian Amazon: *E. oleracea*, *E. precatoria*, *Mauritia flexuosa* (mirití), and *Theobroma grandiflorum* (cupuassu). Targeted LC-QqQ-MS analysis [62] identified 14 flavonoids and 23 phenolic acids, with *M. flexuosa* richest in flavonoids and the *Euterpe* species in phenolic acids; cyanidin-*O*-glucoside was detected exclusively in the latter. Untargeted metabolomic profiling [63] further revealed distinct fingerprints across ripening stages—including cyanidin-*O*-galactoside, isovitexin, quinic acid, and mannitol—highlighting the influence of species, ripening stage, and regional origin on bioactive composition.

Silva et al. [64] evaluated how thermal processing affects a whey–açaí (*Euterpe oleracea*) beverage and verified that heat had little effect on physicochemical traits but significantly reduced phenolics, anthocyanins, and antioxidant capacity, highlighting the heat sensitivity of açaí’s bioactive compounds. Teixeira et al. [65] developed a plant-based ice cream with açaí pulp, jabuticaba peel flour, and faba bean protein, achieving texture and melting properties comparable to conventional dairy açaí cream, thus responding to a growing demand for plant-based and sustainable food alternatives. Martins et al. [66] incorporated pineapple, acerola, and passion fruit waste flour—rich in protein, fiber, and phenolics—into conventional açaí ice cream, achieving high consumer acceptance, particularly for flavor.

Sodré et al. [20] described the creation of a new meat formulation based on the replacement of animal fat by oil extracted from açaí and guar gum-based emulsion gel in goat burgers. This new meat formulation improved the fatty acid profile by reducing saturated fatty acids and the oxidative stability of goat burgers while maintaining sensory quality. Dantas et al. [27] showed that ultrasound enhanced phenolic bioaccessibility, color stability, and enzymatic inactivation in açaí pulp. Collectively, these findings underscore the potential of açaí pulp as a functional ingredient. Maciel et al. [15] developed biodegradable starch films enriched with açaí powder. Those films exhibited improved mechanical and barrier properties while sustaining anthocyanin release, demonstrating the potential of açaí powder for active food packaging.

Deolindo et al. [67] developed and validated an HPLC-DAD method to simultaneously detect and quantify eight prohibited artificial colorants in açaí pulp and commercial products. The method showed high selectivity, sensitivity, and recovery, and was successfully applied to commercial samples, demonstrating its effectiveness for detecting potential product adulteration. Nascimento et al. [68] developed and validated a polymeric ionic liquid-based solid-phase microextraction coupled with GC–MS for pesticide residue monitoring in açaí products. The method was sensitive and accurate, detecting low levels of S-metolachlor in some samples, all well below acceptable daily intake limits. Onça et al. [69] contributed to the analysis of food authenticity by combining attenuated total reflectance—Fourier transform infrared (ATR-FTIR) spectroscopy with chemometric classifiers, achieving high accuracy on the detection of undeclared components in açaí pulp, reinforcing the importance of analytical tools for market trust.

In Health Sciences, studies have focused on both promising benefits and critical safety considerations. Luz et al. [70] confirmed that processed açaí pulp retains its phytochemical richness and antioxidant bioactivity in H9c2 cardiomyocytes and in rat models of CCl4- and menadione-induced oxidative stress. Oliveira et al. [71] showed that açaí seed extract was more effective than moderate exercise training alone in improving cardiovascular health, and their combination yielded the greatest benefits, including improved endothelial function and physical performance.

However, not all findings are uniformly positive: Loubet Filho et al. [72] demonstrated that while açaí intake by obese mice improved cholesterol excretion and short-chain fatty acid production, it also increased adiposity and had no impact on glucose metabolism, underscoring the complexity of metabolic effects. Meanwhile, Godoi et al. [74] developed a stable nanoemulsion containing açaí extract that preserved its antioxidant activity and showed no toxicity in fibroblast cells in vitro, confirming its safety at the cellular level, opening pathways for controlled delivery systems in nutraceuticals and cosmetics. By contrast, Thornton et al. [73] highlighted risks of pharmacological interaction, showing that açaí extracts enhanced the toxicity of methotrexate and tamoxifen in breast cancer cells and normal cell lines, demonstrating the need for caution in clinical trials where açaí extracts are used in combination with drugs such as those described.

The health-related findings summarized above are encouraging but warrant careful contextualization. The large majority of studies in this review rely on in vitro cell-based assays and rodent models, which, while mechanistically informative, do not reliably predict human physiological responses. In vitro antioxidant assays such as DPPH and FRAP measure chemical reactivity under controlled conditions that bear limited resemblance to the complexity of the human gastrointestinal environment [38,39], and extrapolation from murine models to human metabolism is further complicated by differences in pharmacokinetics, gut microbiota composition, and energy homeostasis.

Critically, the available human clinical literature on açaí remains sparse and methodologically heterogeneous: trials differ widely in extract preparation, dosage, duration, and outcome measures, making cross-study comparisons unreliable and dose-response relationships impossible to define at present. The contradictory outcomes observed—such as adiposity increases alongside improved cholesterol excretion [72], or drug toxicity potentiation in combined treatments [73]—further underscore that açaí’s biological effects are context-dependent and cannot be uniformly assumed to be beneficial. Robust, well-powered randomized controlled trials using standardized, commercially representative açaí preparations are an indispensable next step before health-promotion claims can be substantiated for regulatory or public communication purposes.

In Animal Sciences studies demonstrated the value of açaí as a functional feed additive. Silva et al. [76] found improved immune function, hematological parameters, and growth in koi carp, while Bozbay et al. [75] reported enhanced intestinal development, serum lipid profiles, and growth rates in broiler chickens. Together, these results suggest that açaí may serve as a sustainable natural growth promoter in both aquaculture and poultry systems.

Across the studies compiled in Table 3, several cross-cutting methodological trends and shared limitations can be identified. In Food Technology, the widespread adoption of ultrasound-assisted extraction [18,27] and untargeted metabolomics [68] reflects a broader shift toward mechanistically grounded, process-oriented research. A recurring finding is the tension between bioactive preservation and processing requirements: thermal treatments consistently reduce phenolic and anthocyanin content [64], whereas non-thermal alternatives such as ultrasound [27] and high-pressure processing [40] offer superior retention. However, most of these studies are conducted at laboratory scale, and the feasibility of industrial translation remains insufficiently addressed.

In Health Sciences, a convergent finding across preclinical studies is the modulation of oxidative stress and inflammatory pathways by açaí bioactives [70,71], yet results are not uniformly positive—açaí increased adiposity under certain dietary regimes [72] and potentiated the toxicity of anticancer drugs in both cancer and normal cell lines [73]. These contradictory outcomes underscore a shared methodological limitation: the predominance of in vitro and rodent models, the absence of standardized extract preparations, and the lack of dose-response data make cross-study comparisons difficult and clinical extrapolation premature.

In Animal Sciences, despite positive outcomes in poultry [75] and aquaculture [76], studies differ substantially in inclusion rates, species, and outcome measures, preventing definitive recommendations. Overall, the body of recent research confirms açaí’s multifunctional potential but consistently points to the need for standardized experimental protocols, greater use of human clinical trials, and more rigorous pharmacological safety assessments—particularly in contexts involving combined use with medications.

## 6. Potential Applications of Açaí By-Products and Waste Materials

One of the main drawbacks of the açaí industry is the massive generation of waste and by-products, mainly pomace and seeds, which represent up to 85% of the fruit volume [77,78]. Rather than being discarded, however, these residues have increasingly been recognized as valuable raw materials that can be transformed into products with applications in health, food, cosmetics, and energy industries, besides environmental remediation. Açaí seeds, which represent the bulk of residues generated by the açaí industry, are at the forefront of this valorization. Table 4 summarizes key studies on the valorization of açaí by-products and waste materials, highlighting their main applications and potential uses.

In the biomedical field, Arnoso et al. [79] demonstrated that hydroalcoholic açaí seed extracts mitigate intestinal and hypothalamic alterations in obese mice—reducing hyperglycemia, hyperleptinemia, and oxidative stress, while modulating gut microbiota—with effects comparable to metformin. In addition, Oliveira et al. [71] reported that seed extracts ameliorated endothelial dysfunction, vascular hypertrophy, and oxidative stress in hypertensive rats. The combination of açaí seed extracts with exercise training further produced synergistic improvements in lipid profile, vascular reactivity, inflammation, and antioxidant status. Murillo-Franco et al. [80] showed that enzymatic hydrolysis of açaí seeds produces mannooligosaccharides (MOS) in amounts comparable to those from other industrial sources. These MOS, with recognized prebiotic potential, have additional industrial applications, including use as feedstock for biofuels and biochemicals. These studies highlight the versatility of açaí seeds as a valuable byproduct for the bioeconomy, as well as promising tools for the prevention and/or management of metabolic and cardiovascular disorders.

Food applications have also emerged from açaí by-product research. For instance, Campos et al. [81] demonstrated that extracts obtained from processing residues effectively reduced lipid oxidation in beef patties during refrigerated storage, providing a natural alternative to synthetic antioxidants without altering sensory attributes. Silva et al. [82] showed that pomace extracts, after simulated gastrointestinal digestion, retained phenolic acids such as protocatechuic and ferulic acid while preserving strong antioxidant and anti-inflammatory activities, including inhibition of NF-κB and reduction of TNF-α, with no signs of acute toxicity. These results suggest that phenolic stability after digestion may allow absorption and systemic bioactivity, though in vivo confirmation is still needed.

Cosmetic and pharmaceutical applications are equally promising. Zimmer et al. [83] analyzed a standardized seed extract (TI-35) and identified fourteen phenolic constituents with excellent stability and potent antioxidant and anti-aging properties. The extract reduced oxidative stress and metalloproteinase-1 activity in vitro, with no cytotoxicity, skin irritation, or mutagenic effects, confirming its safety for cosmetic applications. In addition, Conrado et al. [84], using Energized Dispersive Guided Extraction (EDGE), obtained phenolic-rich extracts from seeds. These phenolics included especially procyanidins and catechin, two groups of compounds known for their high antioxidant capacity and bioaccessibility, demonstrating the applicability of the extracts obtained by EDGE in dermocosmetics, nutraceuticals, and pharmaceuticals.

Industrial and energy applications have also been demonstrated for açaí residues. Cidreira et al. [85] extracted nanocellulose from açaí bagasse via mixed acid hydrolysis, 2,2,6,6-tetramethylpiperidinyl-1-oxy (TEMPO)-mediated oxidation, and ammonium persulphate oxidation, yielding materials with high crystallinity and thermal stability; oxidative methods additionally produced ionically charged nanofibers with carboxyl groups and improved colloidal stability. Santos et al. [86] showed that drying based on a fluidized bed of agricultural waste enhances energy efficiency and drying kinetics of açaí pulp fragments, external fiber layer, and açaí berry kernels compared with conventional methods, making fluidized bed drying a promising pre-treatment for biofuel production. Farias et al. [78] studied the extraction of cellulose nanofibrils (CNFs) from açaí waste (seeds and fibers) using a combination of alkaline treatment, acid hydrolysis, and ultrasonic processing. The resulting CNFs had high crystallinity, thermal stability, and dispersion stability, making them promising candidates for use in sustainable, green products to replace petroleum-based polymers.

Finally, environmental remediation has emerged as another promising application for açaí. For instance, Lobo et al. [87] demonstrated that both untreated and chemically modified (acid-treated with phosphoric acid and base-treated with sodium hydroxide) açaí seeds serve as efficient biosorbents for removing toxic dyes from wastewater. These biosorbents achieved removal efficiencies above 95% for methylene blue and Remazol brilliant blue R and maintained high activity over several reuse cycles. This application highlights the potential of açaí residues to support low-cost, eco-friendly solutions for industrial effluents while contributing to açaí waste valorization.

When seen together, the above-described applications for açaí waste clearly demonstrate that açaí by-products, whether pomace, seeds, or agricultural residues, are not mere waste but valuable resources. Açaí residues can be transformed into high-value products spanning functional foods, pharmaceuticals, cosmetics, bioenergy, and sustainable adsorbents. This valorization mitigates environmental burdens while expanding the economic and technological significance of the production chain, positioning açaí as a model resource for the circular bioeconomy.

## 7. Challenges in Açaí Research

One of the greatest challenges in açaí research lies in overcoming its high perishability and chemical instability, especially regarding anthocyanins, phenolics, and lipids that are rapidly degraded during processing and storage. In addition, concerns regarding contamination and the safety of açaí consumption have also been raised, as further discussed below.

### 7.1. Açaí Processing

Pioneer works aimed at producing açaí powder demonstrated the influence of drying parameters on product quality. Costa et al. [44] showed that optimized spouted bed drying yielded a stable açaí powder with high anthocyanin retention. Lucas et al. [49] confirmed freeze-drying as the most effective method for preserving anthocyanins, carotenoids, and lipids, a finding corroborated by Oliveira et al. [25], who reported high phenolic content and strong antioxidant potential in freeze-dried açaí pulp. Carneiro et al. [88] provided further evidence for the use of açaí powder as a strategy to extend the fruit’s shelf life, showing that antioxidant activity remained stable for 25 days of storage with no microbiological concern.

More recently, alternative drying methods have been explored to improve açaí pulp preservation. Colaço et al. [56] investigated foam-mat drying and found that it preserved anthocyanin content, moisture, and water activity similarly to freeze-drying, although differences in color parameters were observed. Simão et al. [55] studied conductive thin-film drying and found that, under low pressures, it maintained anthocyanin content and antioxidant activity comparable to freeze-drying, but at the cost of greater lipid oxidation. Meanwhile, Murillo-Franco et al. [89] focused on vacuum-dried encapsulation, which showed effective preservation of anthocyanins and lipids, though light degradation was not fully mitigated. Advances in freeze-dried research in açaí demonstrated that carriers like plant proteins or polydextrose change thermal properties and moisture sorption in freeze-dried powders, demonstrating the complexity of developing shelf-stable açaí ingredients [90].

Thermal and non-thermal processing of juices and pulps also poses significant hurdles. De Jesus et al. [47] found that peroxidase and polyphenol oxidase (PPO) are particularly resistant to high isostatic pressure, with PPO showing only partial inactivation. This is relevant because residual PPO activity can affect cell wall structure, pulp texture, and bioactive compound stability during processing. Oliveira et al. [48] demonstrated that combined non-thermal methods, such as ultrasound and ozonation, improved microbial and enzymatic inactivation but with trade-offs in antioxidant stability. Marangoni Júnior et al. [91] offered a more optimistic perspective, showing that anthocyanins in açaí pulp are thermally stable even under pasteurization-like conditions, with less than 1% degradation under simulated transient processing. Linhares et al. [24], however, highlighted the delicate balance between preserving phenolics and vitamins. For example, while UHT increased anthocyanin bioaccessibility, it markedly reduced vitamin C stability. Non-thermal treatments like High Power Ultrasound (US), UV-pulsed-light, and Low Pressure Plasma (LPP) combined often reduced the bioaccessibility of phenolic compounds, with the US-LPP combination showing the greatest decline. These results emphasize the difficulty of preserving both bioactive compounds and their bioaccessibility during processing.

Encapsulation has been widely reported as a key approach to mitigate chemical instability. Shim et al. [45] demonstrated that nanoencapsulation of açaí concentrate with ascorbic acid and Trolox in chitosan–gum arabic nanoparticles improved antioxidant stability during storage. Dicastillo et al. [92] encapsulated hydroalcoholic freeze-dried açaí extracts into zein electrosprayed particles, achieving thermal resistance under sterilization and baking conditions and improved polyphenol bioaccessibility during digestion.

Innovative formulations and delivery systems have also expanded research frontiers in açaí. Borges et al. [93] characterized açaí seed oil nanoemulsion with strong antioxidant activity and in vitro anticancer effects—inhibiting cell proliferation, migration, and colony formation in HeLa and SiHa cervical cancer cell lines—and no toxicity in vivo, suggesting its potential as a safe therapeutic delivery system. This marks a new phase in açaí research, transitioning from traditional preservation methods to the development of functional delivery systems with targeted therapeutic applications. Finally, Pirozzi et al. [50] tested various açaí extracts in different in vitro models used to demonstrate changes in oxidative stress and lipid metabolism. Results showed that the açaí extracts presented strong antioxidant and hypolipidemic activity, without any evident toxicity. Nevertheless, variability in extract composition, stability, and bioaccessibility continues to complicate the translation of these desired features into standardized products.

The literature highlights ongoing progress in key areas such as the stability of anthocyanins, phenolics, and lipids during drying, storage, and processing; balancing microbial safety with bioactivity preservation; and advancing encapsulation and nanoformulation technologies. Significant strides have been made with innovative drying techniques, enhanced encapsulation methods, and functional delivery systems. While further refinements are possible, these advancements have laid a solid foundation for improving açaí’s stability, scalability, and cost-effectiveness, supporting its broader global adoption as a functional ingredient.

### 7.2. Açaí Contamination by Trypanosoma cruzi

Following the Pan American Health Organization’s (PAHO) declaration of the interruption of vector-borne transmission in the Brazilian Amazon, oral transmission through food contaminated with *Trypanosoma cruzi* has become the predominant route of acute Chagas disease (ACD) [94]. This transmission route accounts for approximately 70–90% of reported acute cases in the region, with more than 95% of orally transmitted infections in Brazil occurring in the Northern Region, particularly in the state of Pará, according to the Brazilian Society of Tropical Medicine [95].

Among the food vehicles associated with oral transmission, *Euterpe oleracea* pulp is the most frequently implicated. A systematic review of 32 ACD outbreaks reported between 1965 and 2022, involving 568 cases and 37 deaths, identified homemade fruit juices—particularly açaí—as the principal source of infection [96]. Notable outbreaks include an 11-case cluster in Barcarena, Pará, in 2006, confirmed through cohort and case–control studies [97], and a 10-case outbreak in Manaus during 2017–2018, in which *T. cruzi* TcIV was molecularly detected in both patient blood samples and the consumed açaí juice [98]. Incidence in Pará peaks during the harvest season (August–November), reflecting a direct temporal link between supply chain activity and disease risk [94].

Contamination occurs mainly when sylvatic triatomines (mainly *Rhodnius pictipes* and *Panstrongylus geniculatus*) or opossum reservoir hosts are co-harvested with açaí fruit and crushed during mechanized processing under inadequate sanitary conditions. *T. cruzi* retains full infectivity in fresh (*in natura*) açaí pulp at room temperature [99], and neither refrigeration nor freezing eliminates this risk [5]. Molecular surveillance has detected *T. cruzi* DNA, including multiple discrete typing units, in commercially sold açaí from Pará and non-endemic markets such as Rio de Janeiro [100], as well as in ready-to-sell açaí pulps from São Paulo State [101]. The concept of “distantiae transmission” describes urban cases in which infected triatomines from harvesting areas are transported with the fruit to urban processing facilities [102]. Recent evidence also suggests that the açaí matrix may modulate host immune responses during oral infection, attenuating pro-inflammatory gastric cytokines while enhancing mucosal protective proteins, with potential implications for disease severity [103].

Regarding preventive measures, pasteurization is considered the most effective. Barbosa et al. [99] demonstrated that heating açaí pulp above 43 °C for at least 20 min inactivates *T. cruzi*, while cooling and freezing alone are insufficient. Industrially pasteurized açaí, required for commercial distribution outside the Amazon and for export, presents near-zero contamination risk. EMBRAPA’s normative chain-wide guidelines cover chlorinated-water washing, pest exclusion, equipment sanitation, and temperature-controlled storage. At the regulatory level, the Brazilian Health Regulatory Agency (ANVISA), through Resolution of the Board of Directors (RDC) nº 218/2005, establishes hygienic-sanitary standards for vegetable-based food preparations, partly motivated by this risk. However, informal artisanal production—where most outbreaks originate—remains largely outside effective regulatory oversight. As global açaí exports expand to Europe, North America, and Asia, the absence of harmonized international standards for *T. cruzi* risk management in açaí represents a growing food safety gap.

## 8. Concluding Remarks and Future Perspectives

This study offers a comprehensive overview of the scientific landscape surrounding açaí (*Euterpe oleracea*), combining bibliometric mapping with evidence from recent research. The bibliometric analysis revealed that global açaí research has consolidated around key themes such as antioxidant activity, phenolic composition, and functional food applications, while expanding into emerging domains including encapsulation technologies, gut microbiota modulation, and innovations in processing and packaging. Although Brazilian institutions remain central to this field, the growing number of international collaborations underscores a clear globalization of açaí research.

The evidence reviewed confirms açaí’s positioning as a multifunctional ingredient: its bioactive richness underpins antioxidant, anti-inflammatory, cardioprotective, and hepatoprotective properties, with emerging evidence for benefits in obesity and hypertension management. Technological innovations—including açaí-enriched dairy alternatives, functional beverages, biodegradable packaging, and reformulated meat products—highlight its versatility and alignment with consumer demand for sustainable and health-promoting foods. Moreover, advances in analytical methodologies have strengthened product authentication and quality assurance, reinforcing consumer confidence and industry integrity.

A persistent challenge for the açaí industry is the large volume of waste generated during pulp processing; however, recent research has demonstrated that these residues—particularly seeds and pomace—can be repurposed into functional ingredients, pharmaceutical and cosmetic actives, biofuels, and eco-friendly materials. Specifically, seed extracts can support metabolic and cardiovascular health, pomace retains antioxidant and anti-inflammatory properties after digestion, and fibrous residues can serve as renewable biosorbents or bio-based polymers.

Several interconnected challenges currently constrain the translation of açaí research into consistent, scalable, and clinically meaningful outcomes. Chief among them is product standardization: raw material quality varies substantially depending on geographic origin, harvesting season, ecotype (*E. oleracea* vs. *E. precatoria*), post-harvest handling, and processing method, yet most studies do not report these variables with sufficient detail to allow meaningful inter-study comparisons—a fundamental barrier to regulatory approval and health claim substantiation. Compounding this, phytochemical concentrations span several orders of magnitude across studies, and the contribution of individual compounds versus synergistic matrix effects remains poorly understood. Bioaccessibility assessment methodologies also lack consensus: static in vitro digestion models, dynamic gastrointestinal simulators, and cell-based transport assays yield substantially different estimates of phenolic bioaccessibility, and no validated protocol has been adopted as a standard for açaí or tropical fruit matrices. Finally, scalability remains a bottleneck: while ultrasound-assisted extraction, nanoencapsulation, supercritical CO_2_ extraction, and high-pressure processing have demonstrated clear advantages at laboratory scale, their economic feasibility and regulatory compliance at industrial scale remain largely undemonstrated—particularly for smallholder and artisanal producers who account for the majority of açaí volume. Addressing these challenges will require coordinated efforts between the research community, industry, and regulatory agencies to develop harmonized protocols and traceability standards throughout the açaí supply chain.

Future research should prioritize well-designed clinical trials to confirm human physiological responses, industrial-scale studies to optimize processing and retention of bioactives, and policy frameworks that encourage sustainable sourcing and international collaboration. By linking scientific progress with industry innovation and regulatory support, açaí can serve as a model bioresource—illustrating how biodiversity-rich species from the Amazon can drive advances in global health, sustainable food systems, and the circular bioeconomy.

## Figures and Tables

**Table 1:** Ranking of leading countries in açaí research (2015–2025) based on VOSviewer analysis of document count.

Country	Documents	Citations	C/D *	TLS **
Brazil	192	3238	16.86	38
USA	23	454	19.74	18
Poland	12	159	13.25	1
Spain	11	149	13.55	8
England	8	271	33.88	6
Colombia	5	103	20.60	1
South Korea	5	85	17.00	0
Canada	4	49	12.25	4
Germany	4	146	36.50	1
China	4	130	32.50	1
Portugal	4	242	60.50	4
Chile	3	47	15.67	2
Italy	3	20	6.67	1
Netherlands	3	103	34.33	4
Türkiye	3	52	17.33	1

* C/D, citations per document; ** TLS, total link strength.

**Table 3:** Most recent studies in açaí from the Web of Science Core Collection *.

Main Area	Article	Journal	Country **	Aim	Results	Key Contribution	Ref.
Food Technology	Development of functional shakes for the Elderly: Utilization of Spirulina biomass and açaí fruit components	Food Bioscience	Brazil	To develop functional powdered shakes enriched with Spirulina biomass, freeze-dried açaí pulp, and açaí seed flour for elderly nutrition.	Formulations enriched with açaí seed flour and pulp increased antioxidant activity, fiber content, and nutritional value without compromising quality.	Functional foods	[61]
	Biorefinery approach for Colombian açaí drupe valorization *(Euterpe oleracea*): physicochemical characterization, anthocyanin extraction, and development of antioxidant-enriched ice cream and cookies	ACS Food Science & Technology	Colombia	To characterize the nutritional composition of Colombian açaí, optimize anthocyanin extraction using ultrasound-assisted extraction, and evaluate its application in developing antioxidant-enriched food products.	Colombian açaí was found to have significant antioxidant and phenolic content, ultrasound-assisted extraction enhanced anthocyanin recovery, and incorporation into cookies showed high acceptability, while ice cream enrichment improved color but faced stability challenges.	Functional foods	[18]
	Flavonoid and phenolic quantification from açaí (*Euterpe oleracea* Mart and *Euterpe precatoria* Mart), mirití (*Mauritia flexuosa* L.), and cupuassu (*Theobroma grandiflorum* (Wild. Ex Spreng.) Schum) from Vaupés, Colombia, using LC-QqQ-MS	Plants	Colombia	To characterize and quantify the flavonoid and phenolic acid profiles of three underexplored Amazonian fruits (*Euterpe oleracea, Euterpe precatoria*, *Mauritia flexuosa*, and *Theobroma grandiflorum*) from Vaupés, Colombia using LC-QqQ-MS and LC-QTOF-MS.	A total of 14 flavonoids and 23 phenolic acid derivatives were detected, with *M. flexuosa* showing the highest total flavonoid content and *E. oleracea/E. precatoria* having the highest phenolic acid levels, with cyanidin-O-glucoside present only in the *Euterpe* species, and overall lower concentrations compared to international reports, likely due to environmental and genetic differences	Functional foods	[62]
	Metabolomic profile of açai (*Euterpe oleracea* Mart., *Euterpe precatoria* Mart.), mirití (*Mauritia flexuosa* L.), and cupuassu (*Theobroma grandiflorum* (Wild. ex Spreng.) Schum) from Colombian Amazon: insights into nutritional composition and ripening dynamics	International Journal of Molecular Sciences	Colombia	To perform a comprehensive metabolomic profiling of three Amazonian fruits (*Euterpe oleracea*, *Euterpe precatoria*, *Mauritia flexuosa*, and *Theobroma grandiflorum*) from the Colombian Amazon to reveal their nutritional composition and changes during ripening using untargeted LC-QTOF-MS and GC-QTOF-MS analyses.	The analysis identified distinct metabolite fingerprints across the fruits and ripening stages—highlighting phenolic compounds like cyanidin-O-galactoside and isovitexin in *Euterpe* species, and dynamic changes in acids and polyols such as quinic acid and mannitol—suggesting their nutritional and functional food potential and the importance of localized metabolomic surveys.	Functional foods	[63]
	Thermal processing effects on antioxidant properties, physicochemical, and sensory characteristics of whey açaí (*Euterpe oleracea* Mart.) beverages	Beverages	Brazil	To evaluate the effects of different thermal processing conditions on the antioxidant properties, physicochemical characteristics, and sensory acceptance of a whey–açaí mixed beverage.	Thermal processing had minimal impact on physicochemical parameters but significantly reduced total phenolic content, total anthocyanins, and antioxidant capacity in proportion to temperature and time, demonstrating the heat sensitivity of bioactive compounds in the beverage.	Functional foods	[64]
	Characterization of the rheological and technological properties of the plant-based ice cream of the açaí and jabuticaba peel flour with faba bean protein	Food Science and Technology International	Brazil	To develop and characterize plant-based ice cream using açaí, jabuticaba peel flour, and faba bean protein.	Higher protein increased hardness and melting resistance; optimal formulation (40% açaí, 8.5% protein, 6.35% oil) achieved properties comparable to dairy ice cream.	Functional foods	[65]
	Physicochemical properties and sensory acceptability of acai sorbet enriched with waste flour from Amazonian fruits	Journal of Food Science and Technology	Brazil	To evaluate the properties of fruit waste flour (FWF) from pineapple, acerola, and passion fruit, and its impact on açaí ice cream.	FWF had high antioxidant content and increased protein and fiber in sorbet-type ice cream, with sensory evaluations showing high consumer acceptance, particularly for flavor.	Functional foods	[66]
	Açai powder-enriched biodegradable starch films: Characterization, release in food simulants and protective effect in photodegradation system	International Journal of Biological Macromolecules	Brazil	To develop starch-based films with açaí powder as an active antioxidant component.	Films showed sustained anthocyanin release, improved mechanical/barrier properties, and protected β-carotene from photodegradation.	Active packaging	[15]
	Açaí pulp treatment in a continuous-loop ultrasonic reactor: effect on enzymatic activity, color, and bioaccessibility of phenolic compounds	ACS Food Science & Technology	Brazil	To evaluate the effects of ultrasonic processing on enzymatic activity, phenolic compounds, and bioaccessibility in açaí pulp.	Continuous processing reduced peroxidase/polyphenol oxidase activity, increased flavonoid and anthocyanin content, improved color, and enhanced phenolic bioaccessibility.	Processing technology	[27]
	Impact of açaí oil and guar gum-based emulsion gel on goat burger quality: Technological, sensory, and fatty acid profile	Meat Science	Brazil	To replace animal fat with açaí oil-based emulsion gel in goat burgers.	Reformulated burgers had lower lipid oxidation, improved fatty acid profile (↑PUFA, ↓SFA), better health indices, and maintained sensory quality with up to 50% replacement.	Meat-based products quality	[20]
	Development and validation of an HPLC-DAD method for the determination of artificial colorants in açaí pulp and commercial products	Food Research International	Brazil	To develop, optimize, and validate a robust HPLC-DAD analytical method for the simultaneous detection and quantification of eight prohibited artificial colorants in açaí pulp and commercial açaí-based products to support authenticity testing and regulatory monitoring	The optimized method showed good selectivity, linearity, low detection limits, and high recovery rates for all targeted dyes and was successfully applied to commercial samples, demonstrating its suitability for detecting potential adulteration of açaí products.	Food authentication	[67]
	Analysis of pesticide residues in açaí-based food products: an approach using polymeric ionic liquid-based solid-phase microextraction coupled to gas chromatography–mass spectrometry	Food Research International	Brazil	To develop and validate a polymeric ionic liquid-based solid-phase microextraction method coupled with gas chromatography–mass spectrometry for analyzing pesticide residues in açaí-based food products	The optimized method showed good sensitivity, linearity, precision, and acceptable recoveries, and detected low levels of S-metolachlor in some samples, with all residues below acceptable daily intake limits, and the approach was practical and environmentally friendly	Food authentication	[68]
	Undeclared components detection in açaí pulp combining ATR-FTIR spectroscopy and one class classifiers	Microchemical Journal	Brazil	To authenticate açaí pulp and detect adulteration with cassava or wheat flour using ATR-FTIR and one-class classifiers.	Both SIMCA and PLSC achieved 100% accuracy in detecting adulteration, confirming ATR-FTIR as a rapid, eco-friendly tool for quality control.	Food authentication	[69]
Health Sciences	Phytochemical profiling of processed açaí pulp (*Euterpe oleracea*) through mass spectrometry and its protective effects against oxidative stress in cardiomyocytes and rats	Antioxidants	Brazil	To characterize processed açaí pulp and evaluate its antioxidant and protective effects in vitro and in vivo.	Identified novel phenolics; pulp extract retained high antioxidant capacity, reduced ROS in cardiomyocytes, and improved antioxidant status in rats, supporting cardioprotective potential.	Cardiovascular Protection	[70]
	Protective effect of açai seed extract (*Euterpe oleracea* Mart.) combined with exercise training on cardiovascular alterations, oxidative stress, and loss of physical performance in spontaneously hypertensive rats	Journal of Medicinal Food	Brazil	To evaluate the protective effects of açaí seed extract (ASE), alone or combined with exercise training, on cardiovascular alterations, oxidative stress, and physical performance loss in spontaneously hypertensive rats.	ASE improved hypertension, vascular function, oxidative stress, and aortic hypertrophy more effectively than exercise alone, while the combination of ASE and exercise provided the greatest overall benefits, including enhanced antioxidant defenses, improved lipid profile, and restored physical performance.	Cardiovascular Protection	[71]
	The effects of açaí (*Euterpe oleracea*) intake on gut bacteria and their metabolites in obese mice	Journal of Functional Foods	Brazil	To evaluate the prebiotic and metabolic effects of dietary açaí pulp in obese mice.	Açaí increased SCFA production and fecal cholesterol excretion but also led to higher adiposity and had limited benefits on glucose metabolism.	Gut microbiota	[72]
	Açaí extract and anticancer drug combination promotes synergistic toxicity and apoptosis in MCF-10A cells of breast cancer model	Journal of Ethnopharmacology	USA	To evaluate the toxicological interactions between açaí extracts and common anticancer drugs (methotrexate and tamoxifen) in breast cancer and normal breast epithelial cells.	Açaí extracts potentiated the toxicity of both drugs across all tested cell lines. Methanol and acidic methanol extracts combined with methotrexate caused strong synergy, significantly increasing toxicity in normal MCF-10A cells and inducing apoptosis. Methotrexate potentiation was also observed in MCF-7 cancer cells, while tamoxifen toxicity was enhanced in MCF-7 and in pre-exposed MCF-10A cells.	Drug interaction risk	[73]
	Açaí-loaded nanoemulsion: synthesis, characterization, and in vitro safety profile	Applied Sciences	Brazil	To produce and evaluate the stability and safety of nanoemulsions with açaí extract.	A stable nanoemulsion was obtained at 4 mg/mL of açaí extract, showing favorable physicochemical stability under refrigeration. In vitro assays confirmed non-toxicity in fibroblasts, with preservation of cellular viability, DNA integrity, and antioxidant potential.	Nanoemulsion delivery	[74]
Animal Sciences	Growth performance and carcass, gastrointestinal tract, meat quality, and serological properties of broiler chickens fed diets supplemented with tropical açaí (*Euterpe oleracea*) fruit extract	Tropical Animal Health and Production	Türkiye	To assess açaí fruit extract as a feed additive in broiler diets.	Using 5 g/kg supplementation improved growth performance, carcass traits, intestinal development, and serum lipid profiles, without compromising meat quality.	Poultry nutrition	[75]
	Dietary supplementation with açai (*Euterpe oleracea*) improved the haemato-immunological parameters and growth performance of koi carp (*Cyprinus carpio* Linnaeus, 1758)	Veterinary Research Communications	Brazil	To evaluate the effects of dietary açaí supplementation on growth and immune responses of koi carp.	Optimal inclusion (15 g/kg) improved growth, feed conversion, immune activity, and antimicrobial response, showing potential as a natural aquaculture additive.	Aquaculture feed	[76]

* As of 31 December 2025. ** Country of the main author. Ref., reference.

**Table 4:** Summary of studies on valorization of açaí (*Euterpe oleracea*) by-products and waste materials.

Application Category	By-Product	Aim	Key Findings	Potential Applications	Ref.
Biomedical	Seeds	To evaluate the effect of seed hydroalcoholic extract on obesity-related intestinal and hypothalamic alterations in mice	Reduced hyperglycemia, hyperleptinemia, oxidative stress, and modulated gut microbiota; effects comparable to metformin.	Nutraceuticals/Metabolic health	[79]
Biomedical	Seeds	To investigate the combined effect of seed extract and exercise on cardiovascular health in hypertensive rats	Improved endothelial function, reduced vascular hypertrophy and oxidative stress; synergistic benefits with exercise training.	Nutraceuticals/Metabolic health	[71]
Biomedical/Bioenergy	Seeds	To optimize the enzymatic hydrolysis of seeds for mannooligosaccharide (MOS) production	MOS yields comparable to other industrial sources; prebiotic potential and applicability as biofuel feedstock.	Prebiotics/Biofuels/Biochemicals	[80]
Food Technology	Pomace	To assess the use of residue extracts as natural antioxidants in beef patties	Effectively reduced lipid oxidation during refrigerated storage without altering sensory attributes.	Active food ingredients/Meat preservation	[81]
Food Technology/Nutraceuticals	Pomace	To evaluate the bioaccessibility and biological activity of pomace extracts after simulated GI digestion	Retained protocatechuic and ferulic acids; maintained antioxidant and anti-inflammatory activity (NF-κB, TNF-α inhibition); no acute toxicity.	Functional foods/Nutraceuticals	[82]
Cosmetics/Pharmaceuticals	Seeds	To characterize and evaluate the efficacy of a standardized seed extract for cosmetic use	14 phenolic constituents identified; antioxidant and anti-aging activity; reduced MMP-1 activity; no cytotoxicity, skin irritation, or mutagenicity.	Cosmetics/Anti-aging ingredients	[83]
Cosmetics/Pharmaceuticals	Seeds	To optimize phenolic extraction from seeds using Energized Dispersive Guided Extraction (EDGE)	Rich in procyanidins and catechin, with high antioxidant capacity and bioaccessibility.	Dermocosmetics/Nutraceuticals/Pharmaceuticals	[84]
Industrial/Materials	Bagasse (seeds + fibers)	To extract nanocellulose from açaí bagasse via mixed acid hydrolysis and oxidative methods	Nanocellulose with high crystallinity and thermal stability; oxidative methods yielded charged nanofibers with improved colloidal stability.	Sustainable materials/Bio-based polymers	[85]
Industrial/Bioenergy	Pulp fragments, fiber, kernels	To optimize fluidized bed drying of açaí agricultural waste for bioenergy pre-treatment	Enhanced energy efficiency and drying kinetics compared to conventional methods.	Bioenergy/Biofuel pre-treatment	[86]
Industrial/Materials	Seeds + fibers	To extract cellulose nanofibrils (CNFs) using alkaline treatment, acid hydrolysis, and ultrasound	CNFs with high crystallinity, thermal stability, and dispersion stability; suitable to replace petroleum-based polymers.	Sustainable materials/Green products	[78]
Environmental Remediation	Seeds (untreated and chemically modified)	To evaluate the use of açaí seeds as biosorbents for toxic dye removal from wastewater	>95% removal efficiency for methylene blue and Remazol brilliant blue R; stable over multiple reuse cycles.	Wastewater treatment/Industrial effluents	[87]

MMP-1, matrix metalloproteinase-1; MOS, mannooligosaccharides; CNFs, cellulose nanofibrils; GI, gastrointestinal; NF-κB, nuclear factor kappa B; TNF-α, tumor necrosis factor alpha.

## Data Availability

No new data were created or analyzed in this study.

## Supplementary Materials

The following supporting information can be downloaded at: https://www.mdpi.com/article/10.3390/foods15122203/s1, Figure S1: PRISMA flow diagram adapted for the narrative review on *Euterpe oleracea* (açaí); Figure S2: Leading institutions on açaí from Bibliometrix analysis (upper image) and connections between organizations from VOSviewer analysis using data from the Web of Science Core Collection (lower image); Figure S3: Trend topics over the years (2015–2025) related to açaí berry products as identified through Bibliometrix analysis using data from the Web of Science Core Collection; Table S1: Overview of the dataset on *Euterpe oleracea* (açaí) berry products retrieved from the Web of Science Core Collection and analyzed using Bibliometrix via the Biblioshiny interface; Table S2: Keywords from clusters generated through VOSviewer analysis of 245 articles on the use of açaí (*Euterpe oleracea*) and its derived products; Table S3: Ranking of leading organizations in açaí research (2015–2025) as per VOSviewer analysis of document count.
