# Supplementary material for: Bibliometric Insights and Recent Advances in the Science, Technology, and Sustainability of Açaí (Euterpe oleracea) from Amazonian Staple to Global Superfruit
Source: Foods. 2026 Jun 18;15(12):2203. doi: 10.3390/foods15122203 (PMC13298187; doi:10.3390/foods15122203)
Supplement: Supplementary file 1 [file foods-15-02203-s001.zip › foods-4360880-supplementary.pdf]

Supplementary Material

Bibliometric insights and recent advances in the science, technology, and sustainability of açaí (*Euterpe oleracea*) from Amazonian staple to global superfruit

Adriano Cezar Delphim [1], Gerson Lopes Teixeira [1,2], Adaucto Bellarmino Pereira-Netto [1]

[1] Graduate Program in Food Engineering, Department of Chemical Engineering, Federal University of Paraná, 82590-300, Curitiba, PR, Brazil

[2] Graduate Program in Food and Nutrition, Federal University of Paraná, 80210-170, Curitiba, Paraná, Brazil

Table S1 Overview of the dataset on *Euterpe oleracea* (açaí) berry products retrieved from the Web of Science Core Collection and analyzed using Bibliometrix via the Biblioshiny interface.

**MAIN INFORMATION ABOUT DATA**	
Timespan	2015:2025
Sources (Journals, Books, etc.)	124
Documents	245
Annual Growth Rate %	3.51
Document Average Age	5.73
Average citations per doc	17.73
**DOCUMENT CONTENTS**	
Keywords Plus (ID)	809
Author's Keywords (DE)	810
**AUTHORS**	
Authors	1336
Authors of single-authored docs	0
**AUTHORS COLLABORATION**	
Single-authored docs	0
Co-Authors per Doc	6.91
International co-authorships %	23.67
**DOCUMENT TYPES**	
Article	241
Article; early access	2
Article; proceedings paper	2

Table S2 Keywords from clusters generated through VOSviewer analysis of 245 articles on the use of açaí (*Euterpe oleracea*) and its derived products.

Cluster	#	Keywords
Cluster 1	19	Amazon palm berry; antioxidant; antioxidant capacity; berry; chemistry; *Euterpe oleracea* Mart.; extract; flavonoids; inflammation; insulin-resistance; metabolism; obesity; oxidative stress; phytochemical compounds; pigment stability; pulp; quantification; rats; toxicity
Cluster 2	15	Acid; antioxidant activity; antioxidants; capacities; capacity; DPPH; *Euterpe oleracea*; extracts; functional foods; impact; lipid oxidation; mechanisms; phenolic compounds; phenolics; polyphenols
Cluster 3	14	Açaí; açaí pulp; Chagas disease; consumption; DNA; extraction; food analysis; *in vitro*; oral transmission; outbreak; texture; *Trypanosoma cruzi*; viability
Cluster 4	14	Açaí oil; anthocyanin; *Euterpe oleracea*; *Euterpe oleracea* Mart.; foods; health; milk; oil; optimization; phenolic compounds; physical properties; stability; supplementation; temperature
Cluster 5	14	Anthocyanins; ascorbic acid; bioactive compounds; carotenoids; chemometrics; color; fruit; identification; L.; pasteurization; physicochemical properties; thermal processing; total antioxidant capacity
Cluster 6	12	Açaí berry; antioxidant properties; assay; bioaccessibility; chemical composition; encapsulation; films; food; fruits; Mart.; quality; seeds

#number of keywords

Table S3 Ranking of leading organizations in açaí research (2015-2025) as per VOSviewer analysis of document count.

Organization[1]	Country	Documents	Citations	TLS***
Univ Estadual Campinas	Brazil	21	506	10
Univ Sao Paulo	Brazil	19	230	14
Univ Fed Ceara	Brazil	17	399	8
Univ Campinas Unicamp	Brazil	12	165	9
Fed Univ Para	Brazil	11	272	10
Univ Fed Para	Brazil	11	97	8
Univ Fed Vicosa	Brazil	10	170	6
Univ Fed Rio de Janeiro	Brazil	9	203	6
Univ Fed Santa Maria	Brazil	9	92	5
Univ Fed Santa Catarina	Brazil	8	119	9
Univ Fed Amazonas	Brazil	7	271	4
Texas A&M Univ	USA	6	198	3
Univ Fed Ouro Preto	Brazil	6	109	4
Univ Fed Paraiba	Brazil	6	206	3
Univ Fed Rio Grande do Sul	Brazil	6	88	5
Embrapa Amazonia Oriental	Brazil	5	165	6
Fed Univ Rio Grande	Brazil	5	107	0
Univ Fed Amapa	Brazil	5	102	4
Univ Fed Sao Joao del Rei	Brazil	5	74	6
Univ Fed Uberlandia	Brazil	5	71	8
Embrapa Agroind Trop	Brazil	4	76	4
Franciscan Univ	Brazil	4	46	5
Fundacao Oswaldo Cruz	Brazil	4	45	1
Inst Food Technol ITAL	Brazil	4	73	5
Univ Estado Rio de Janeiro	Brazil	4	145	6
Univ Fed Lavras	Poland	4	92	1
Univ Fed Parana	Brazil	4	29	4
Univ Reading	UK	4	193	2

[1]The data and names are presented abbreviated as retrieved from VOSviewer.*Universidade Estadual de Campinas. **Universidade Federal do Pará. ***Total Link Strength

Supplementary Figures

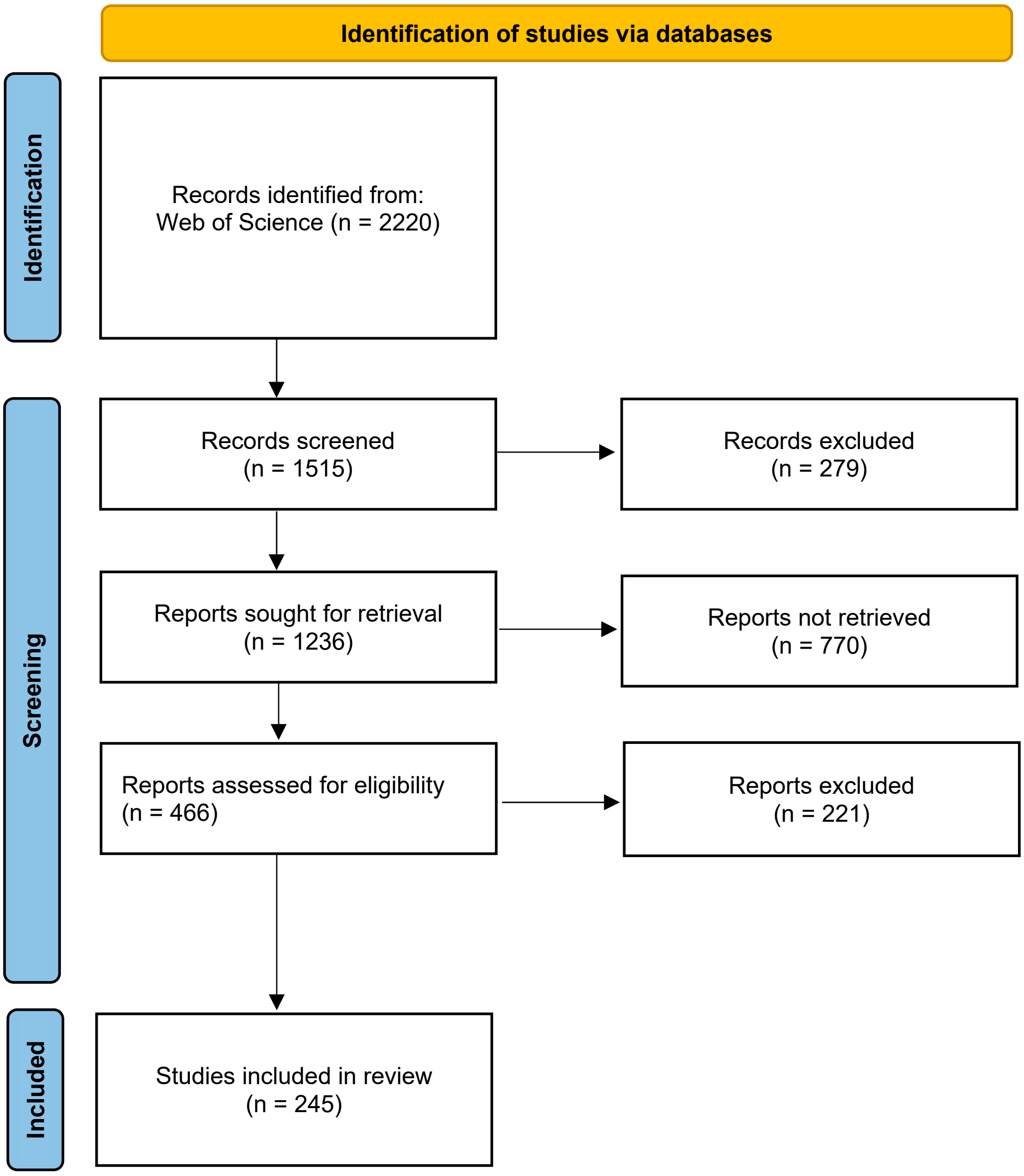

Figure S1 PRISMA flow diagram adapted for the narrative review on *Euterpe oleracea* (açaí)

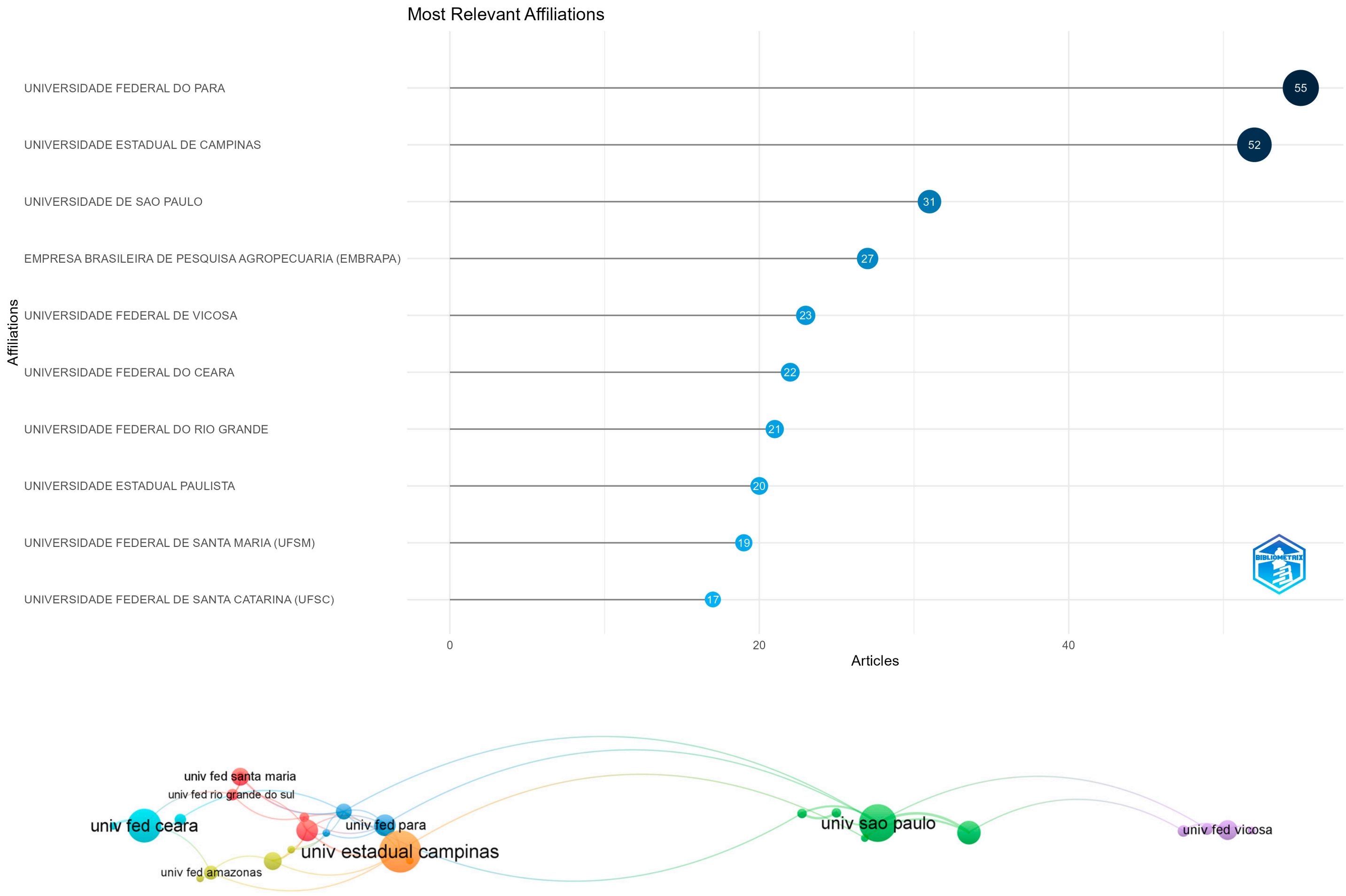

Figure S2 Leading institutions on açaí from Bibliometrix analysis (upper image) and connections between organizations from VOSviewer analysis using data from the Web of Science Core Collection (lower image).

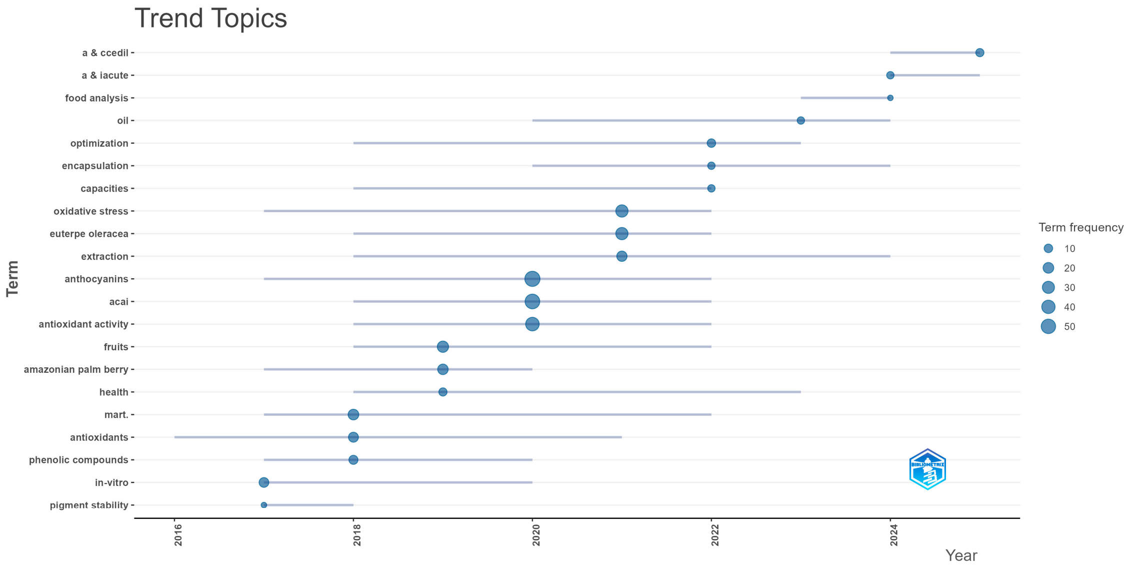

Figure S3 Trend topics over the years (2015-2025) related to açaí berry products as identified through Bibliometrix analysis using data from the Web of Science Core Collection.
